# Quantitative evaluation of contrast agent uptake in standard fat‐suppressed dynamic contrast‐enhanced MRI examinations of the breast

**DOI:** 10.1002/mp.12652

**Published:** 2017-11-30

**Authors:** Evanthia Kousi, Joely Smith, Araminta E. Ledger, Erica Scurr, Steven Allen, Robin M. Wilson, Elizabeth O'Flynn, Romney J.E. Pope, Martin O. Leach, Maria A. Schmidt

**Affiliations:** ^1^ CR‐UK and EPSRC Cancer Imaging Centre Royal Marsden NHS Foundation Trust Institute of Cancer Research Sutton Surrey SM2 5PT UK; ^2^ Department of Radiology Royal Marsden NHS Foundation Trust Chelsea, London SW3 6JJ UK; ^3^ Brighton and Sussex University Hospitals NHS Trust Brighton BN2 5BE UK

**Keywords:** breast, breast cancer, calibration, DCE‐MRI, quantification

## Abstract

**Purpose:**

To propose a method to quantify T_1_ and contrast agent uptake in breast dynamic contrast‐enhanced (DCE) examinations undertaken with standard clinical fat‐suppressed MRI sequences and to demonstrate the proposed approach by comparing the enhancement characteristics of lobular and ductal carcinomas.

**Methods:**

A standard fat‐suppressed DCE of the breast was performed at 1.5 T (Siemens Aera), followed by the acquisition of a proton density (PD)‐weighted sequence, also fat suppressed. Both sequences were characterized with test objects (T_1_ ranging from 30 ms to 2,400 ms) and calibration curves were obtained to enable T_1_ calculation. The reproducibility and accuracy of the calibration curves were also investigated. Healthy volunteers and patients were scanned with Ethics Committee approval. The effect of B_0_ field inhomogeneity was assessed in test objects and healthy volunteers. The T_1_ of breast tumors was calculated at different time points (pre‐, peak‐, and post‐contrast agent administration) for 20 patients, pre‐treatment (10 lobular and 10 ductal carcinomas) and the two cancer types were compared (Wilcoxon rank‐sum test).

**Results:**

The calibration curves proved to be highly reproducible (coefficient of variation under 10%). T_1_ measurements were affected by B_0_ field inhomogeneity, but frequency shifts below 50 Hz introduced only 3% change to fat‐suppressed T_1_ measurements of breast parenchyma in volunteers. The values of T_1_ measured pre‐, peak‐, and post‐contrast agent administration demonstrated that the dynamic range of the DCE sequence was correct, that is, image intensity is approximately directly proportional to 1/T_1_ for that range. Significant differences were identified in the width of the distributions of the post‐contrast T_1_ values between lobular and ductal carcinomas (*P* < 0.05); lobular carcinomas demonstrated a wider range of post‐contrast T_1_ values, potentially related to their infiltrative growth pattern.

**Conclusions:**

This work has demonstrated the feasibility of fat‐suppressed T_1_ measurements as a tool for clinical studies. The proposed quantitative approach is practical, enabled the detection of differences between lobular and invasive ductal carcinomas, and further enables the optimization of DCE protocols by tailoring the dynamic range of the sequence to the values of T_1_ measured.

## 1 Introduction

Dynamic Contrast‐Enhanced MRI (DCE‐MRI) is a powerful clinical tool for the detection, diagnosis, and staging of the breast cancer.^1^, ^2^ Following the administration of a gadolinium chelate contrast agent, tissue perfusion can be estimated using changes in the signal intensity over time on a series of images obtained with fast 3D T_1_‐weighted pulse sequences. Clinical DCE‐MRI examinations of the breast are commonly performed with fat suppression and provide time‐signal intensity curves to qualitatively assess the enhancement kinetics of the contrast agent uptake in tumors. In contrast, pharmacokinetic modeling offers a quantitative approach to investigate tumor vascularity associated with malignancy and has been shown to improve the diagnostic performance of MRI as well as the prediction of treatment response.^3^, ^4^, ^5^ In this setting, a separate proton density sequence is used as a reference, enabling the calculation of T_1_. Pharmacokinetic modeling requires rapid data acquisitions, sacrificing spatial resolution and breast coverage, and is not currently a part of the standard clinical practice.

The breast MRI is recommended for the assessment of biopsy‐proven invasive lobular carcinomas (ILCs). This is due to the lower diagnostic performance of other imaging modalities in accurately defining the extent of disease,^6^, ^7^ which can be caused by the diffuse growth pattern of some ILCs. Differences in the DCE‐MRI enhancement characteristics between ILCs and invasive ductal carcinomas (IDCs) have been previously demonstrated.^8^, ^9^

In this work, we propose to combine a high‐resolution fat‐suppressed clinical DCE‐MRI sequence with the ability to perform quantitative T_1_ measurements by introducing a proton density‐weighted sequence as a reference. We demonstrate our method by comparing the enhancement characteristics of two groups of breast patients: ILCs and IDCs. In addition, we evaluate the accuracy and reproducibility of the obtained T_1_ values. Furthermore, we show that the dynamic range of our DCE‐MRI sequence is suited to the range of T_1_ values measured in our clinical breast examinations, enabling contrast agent uptake to be correctly depicted.

## 2 Methods

### 2.A. Imaging protocol

Subjects were scanned at 1.5 T (Aera, Siemens, Erlangen, Germany) using an eight‐channel breast receiver coil. Volunteers were scanned with an approval of the Research Ethics Committee (UK NHS HRA/NRES Committee London‐Chelsea 1406/18‐06‐1997) and written consent was obtained. The retrospective analysis of patient studies was approved by the Research Ethics Committee (“Evaluation of Breast MRI Protocols”, Service Evaluation). A standard clinical breast DCE‐MRI protocol was performed using spectrally selective (Spectral Attenuated Inversion Recovery, SPAIR) pulses for fat suppression and three‐dimensional (3D) T_1_‐weighted spoiled gradient‐echo sequences (TE/TR = 2/4.5 ms, flip angle = 18°, pixel size = 1.31 × 1.31 × 1 mm, parallel imaging factor 2, number of slices = 160, acquisition matrix = 290 × 320, FOV = 380 × 420 mm^2^). The readout gradient direction was anterior/posterior to minimize cardiac motion artifacts over the breasts. A single dose of contrast agent (Dotarem, Guerbet, France) was administered at 2–3 mL/s (MedRad,USA) depending on the size of the largest vascular access device that could be fitted to the patient. One pre‐ and eight post‐contrast transaxial 3D data sets were acquired in 56 s each, in agreement with the current national guidelines.^10^ A proton density (PD)‐weighted sequence was subsequently obtained with identical parameters to the DCE‐MRI sequence using a lower flip angle of 4°.

### 2.B. Quantitative assessment of contrast agent uptake

In quantitative DCE examinations undertaken for pharmacokinetic modeling, T_1_ is calculated from a combination of the two data sets obtained with differing amounts of T_1_ weighting (spoiled gradient echoes with high and low flip angles).^11^ The sequence with the high flip angle is T_1_ weighted while the sequence with low flip angle has minimal T_1_ weighting. The concentration of contrast agent in each voxel is calculated quantitatively from T_1_ values:

(1) $$\left[\mathrm{Gd}\right]=\left(1/T_{1}-1/T_{1\;\mathrm{pre}}\right)/r$$

where T_1pre_ is the native T_1_ of the tissue and r is the relaxivity of the administered contrast agent.

Although an analytical solution of the Bloch equations is not usually practical for the fat‐suppressed spoiled gradient‐echo sequences used in DCE, these sequences follow the same principles and both experimental work and numerical simulations demonstrate fat‐suppressed and non‐fat‐suppressed sequences have similar contrast characteristics: image intensity should be proportional to 1/T_1_ over the T_1_ range of interest.^12^ Therefore, subtracting a post‐contrast image (S_1_) from the pre‐contrast baseline (S_pre_) demonstrates the contrast agent uptake qualitatively. However, even in a perfect system with no changes in image intensity associated with imperfect excitation or receiver coil sensitivity, quantitative analysis is not possible because there will be (a) variations of image intensity in the same examination associated with differences in the proton density (equilibrium magnetization) from voxel to voxel and (b) changes to coil filling factor and changes to system gain which makes it impossible to compare directly an examination to another or a patient to another.

It is possible to define an enhancement ratio (ER) as:

(2) $$\mathrm{ER}=\left(S_{1}-S_{\mathrm{pre}}\right)/S_{\mathrm{pre}}$$

As a ratio, ER is not affected by proton density or by coil sensitivity, but is affected by the native T_1_ value. Variations in ER within a lesion may not relate to different contrast agent uptake, but simply to different native T_1_ values; the same applies to longitudinal changes in a patient study. ER is only semiquantitative. This is the main motivation to develop a method to calculate T_1_ using fat‐suppressed sequences.

To calculate T_1_, we introduce a low flip angle image which is practically proton density weighted and also fat suppressed. Using two sequences with different T_1_ weighting, we calculate T_1_ post‐contrast for each pixel. For that purpose, the behavior of each sequence is studied with a test object comprising a very wide range of T_1_ values, and empirical curves are used, instead of the solution of the Bloch equations.^11^

In a similar way to pharmacokinetic studies, we related the DCE/PD image ratio to the T_1_ value using an experimental measurement on test objects to provide a calibration curve. This method provided a direct measurement of the T_1_ value at the end of the DCE protocol; the last frame of the DCE and the PD acquisition are used jointly to calculate T_1_ (T_1post_). This approach presumes a slow clearance of the contrast agent from the patient's system between the last DCE and PD acquisitions, and therefore, no significant changes in the contrast agent concentration are expected over those few minutes. In order to calculate T_1_ values in previous frames, we used the same principles employed in quantitative DCE examinations (without fat suppression); we presumed that all changes of image intensity were associated with T_1_ (i.e., there was no change to the equilibrium magnetization; there were no other hardware changes; and the signal intensity for DCE was a known function of T_1_).

### 2.C. Test objects

Plastic tubes filled with aqueous solutions of CuSO_4_ of different concentrations were used to generate the calibration curves. Standard Inversion Recovery (IR) measurements were employed to provide the reference values for this test object, comprising T_1_ values within the range 30 ms–2,600 ms. These solutions were scanned with the DCE and PD sequences to produce a curve representing the ratio between the image intensity obtained with DCE and PD images, now referred to as image ratio, as a function of R_1_ = 1/T_1_. A separate curve was calculated to provide calibrated image intensity values for the imaging sequences also as a function of R_1_. A least‐square smooth spline line fitting was performed using R statistical software (R v.3.0.2, www.r-project.org) and the % coefficient of variation was used to evaluate the reproducibility of the calibration curves. The DCE pulse sequence employed has a short TE, and therefore, we do not expect the contrast to have T_2_—or $T_{2}^{*}$—weighting for the test objects used in calibration and for the breast.

This measurement was repeated on two different occasions separated by 4 months to calibrate the T_1_ measurement and to evaluate the stability of the calibration curves. For this measurement, the power applied by the fat suppression SPAIR pulse was set to zero, and therefore, the calibration curves were not affected by B_0_ inhomogeneity.

The effect of B_0_ inhomogeneity was investigated separately, with the same set of solutions. The central frequency was changed in four steps of 50 Hz in both directions (−200 Hz < ω_0_ < 200 Hz), and therefore, the applied fat suppression pulse partially suppressed water signals. Errors in T_1_ measurements were attributed to off‐resonance effects. In addition, a uniform test object (T_1_~110 ms) was scanned with the same sequences to verify whether the ratio between DCE and PD images was constant over the breast volume, as this could be affected by B_1_ inhomogeneity^13^ and uniformity filters.^14^ The percent ratio image uniformity (PRIU) was calculated over the coil volume to be occupied by the breasts according to the following equation:

(3) $$PRIU=100\;\times \;\left(1-\frac{Ratio_{\max}-Ratio_{\min}}{Ratio_{\max}+Ratio_{\min}}\right)$$

### 2.D. Clinical examinations

The DCE examinations of 20 patients with histologically confirmed breast tumors were analyzed. Tumors comprised ten lobular carcinomas [six grade 2 ILCs, four lobular carcinomas in situ (LCIS)] and ten ductal carcinomas [two grade 1, five grade 2, and one grade 3 IDCs, two high‐grade ductal carcinomas in situ (DCIS)]. All post‐contrast 3D data sets were registered to the pre‐contrast data set prior to analysis using a rigid registration method with six degrees of freedom (3D Slicer v. 4.4.0, www.slicer.org). The largest transaxial cross section for each tumor was chosen and the tumor outline performed using in‐house software (IDL 8.4 Boulder, CO, USA) and was approved by a specialist breast Radiologist.

In addition, DCE and PD data sets were obtained for two healthy volunteers by changing the central frequency in steps of 25 Hz in order to investigate further the off‐resonance effects on clinical T_1_ calculations. The fibroglandular tissue was segmented using the k‐means clustering algorithm over the entire breast volume (IDL 8.4 Boulder, CO, USA).

The T_1_ relaxation time of the tumors was calculated on a pixel‐by‐pixel basis using the test object calibration curves at three time points: (a) before contrast administration (T_1pre_), (b) at peak‐contrast uptake, that is, the shortest T_1_ (T_1peak_), and (c) the post‐contrast (final) frame of the DCE examination (T_1post_). In cases of late tumor enhancement, T_1peak_ = T_1post_. Gadolinium concentration [Gd] and the %ER of the tumors were also calculated pixel‐by‐pixel at peak‐ and post‐enhancement frames, using Eqs. (1) and (2), respectively. The following characteristics of lobular and ductal carcinomas were compared: peak‐enhancement frame, median, and interquartile range (IQR) of the T_1_ values at the pre‐, peak‐, and the post‐enhancement frames, and of [Gd] and %ER at peak‐ and post‐enhancement. The Wilcoxon rank‐sum test was used for statistical analysis with a significance level of *P* < 0.05 (R v.3.0.2, www.r-project.org).

## 3 Results

### 3.A. Test object study

**Figure 1:**
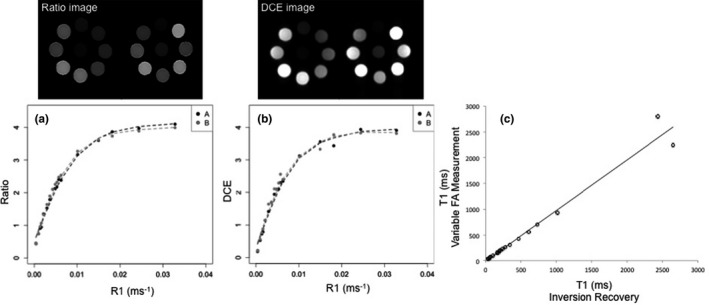
Calibration curves as measured in two separate occasions, A (black) and B (grey): ratio (a) and DCE (b) image intensity as a function of R_1_ (ms^−1^) for test objects with T_1_ ranging from 30 ms to 2600 ms. (c) Fat‐suppressed T_1_ measurements agree with the IR measurements for the test objects.

Figure 1 shows the calibration curves produced using phantom data for the clinical pulse sequences on two separate occasions. Calibration curves were produced for the ratio image intensity [Fig. 1(a)] and the DCE image intensity [Fig. 1(b)]. The image intensity of the DCE sequence can be considered approximately directly proportional to R_1_ for R_1_ < 0.01 ms^−1^ (or T_1_ > 100 ms). The calibration curves proved to be reproducible: the calculated coefficient of variation for different T_1_ values varied from 0.1% to 9% for the ratio image and 0.5%–10% for the DCE image. Fat‐supressed T_1_ measurements were in agreement with IR measurements for the test objects [Fig. 1(c)]: the average absolute difference between fat‐suppressed T_1_ measurements and IR measurements of the test tube solutions was 7% (range 0.28%–16%); the largest difference was found for the longest T_1_ (2600 ms).

**Figure 2:**
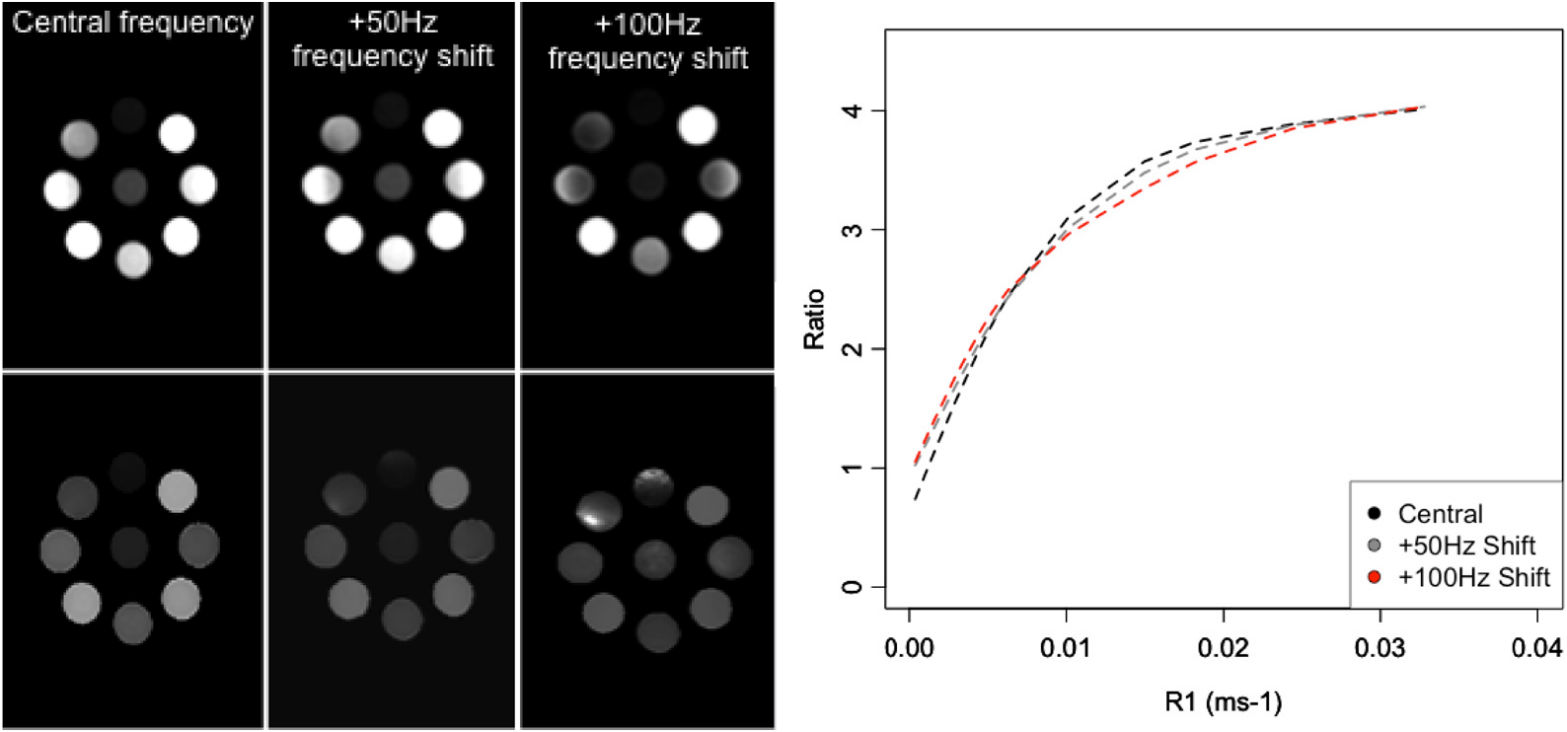
Test objects (T_1_ range 30–2400 ms) DCE (top row) and ratio (bottom row) images acquired with different resonance frequencies showing errors being introduced by a gradual suppression of the water signal resulting in distorted calibration curves (right). [Color figure can be viewed at wileyonlinelibrary.com]

The curves in Figs. 1(a) and 1(b) are affected by off‐resonance effects if field inhomogeneity causes the fat suppression pulse to suppress water. Frequency shifts under 50 Hz introduced changes up to 19% to T_1_ measurements in the range 100 ms < T_1_ < 1000 ms. Figure 2 shows DCE and ratio images of tube solutions, with T_1_ ranging from 30 ms to 2400 ms, as a function of frequency shift demonstrating a progressive suppression of water signal that results in distorted calibration curves. In volunteer studies, T_1_ values obtained for normal breast parenchyma showed small variations (< 3%) for frequency shifts below 50 Hz. The T_1_ of the breast parenchyma was also measured on a set of patients with unilateral disease (Appendix 1).

**Figure 3:**
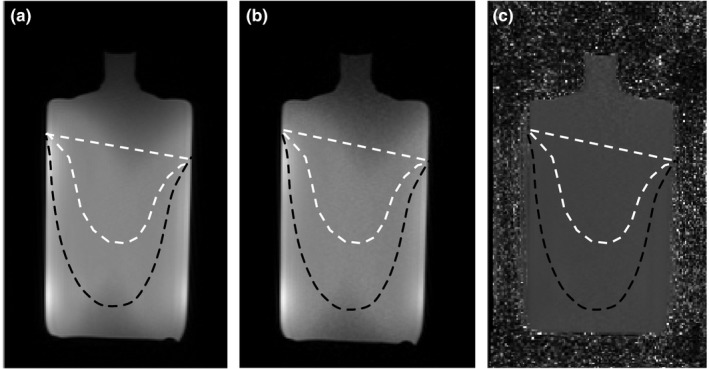
Transaxial slice at the center of a uniform test object acquired with DCE (a) and PD sequences (b) and the corresponding ratio image (c). The right side of the breast coil is shown. High intensity uniformity of the ratio image is demonstrated over the coil area that is occupied by the breast. Dashed lines show the area at the center of the breast coil that is occupied by a small (white dashed line) or large (black dashed line) breast.

Figure 3 shows a transaxial slice of a uniform test object acquired with DCE [Fig. 3(a)] and PD [Fig. 3(b)] sequences and the ratio between them [Fig. 3(c)]. Ratio image is uniform over the coil volume that is occupied by small or large breasts (PRIU_Right Coil_ = 93% and 87%, respectively, PRIU_Left Coil_ = 91% and 90%, respectively), suggesting only relatively small errors associated with spatial variations in B_0_ and B_1_.

### 3.B. Clinical study

**Figure 4:**
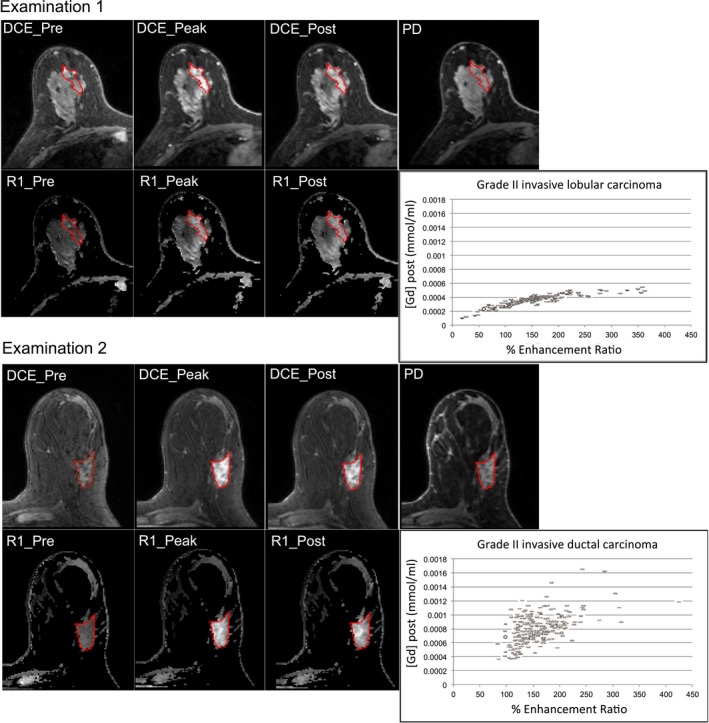
Pre‐, peak‐, and post‐contrast enhancement images followed by the corresponding PD images and R_1_ maps of a grade II invasive lobular carcinoma (Examination 1) and grade II invasive ductal carcinoma (Examination 2); the mean native T_1_ ± standard deviation for examinations 1 and 2 is 1311 ± 298 ms and 750 ms ± 139 ms, respectively. Graphs relate the % Enhancement ratio and [Gd] for these examinations. Only in the first example, the [Gd] rises monotonically with the Enhancement Ratio, in the second example, a more complex relationship is mediated by variations in native T_1_. R_1_ maps for examinations 1 and 2 have been scaled independently. Within individual examinations, R_1_ maps have been scaled equally. [Color figure can be viewed at wileyonlinelibrary.com]

Figure 4 shows the pre‐, peak‐, and the post‐contrast enhancement frames of two breast examinations, followed by the image with low flip angle (PD) and the corresponding R_1_ measurements using the test object calibration curves. The % ER was also calculated pixel‐by‐pixel using Eq. (2) and compared with [Gd] post‐enhancement [Eq. (1)]. The graphs show that although both cases have the same ER range, the range of values for the gadolinium concentration is different. Axes have been scaled equally to highlight this observation.

Median T_1_, [Gd], and %ER values were calculated for every patient; mean ± standard deviation is shown in Table 1. There were no significant differences between the two cancer groups (*P* > 0.05, *P* values in Table 1). Peak‐ enhancement occurred in the final frame for four (three ILC and one LCIS) of ten lobular carcinomas suggesting slower uptake of the contrast agent for these tumors. Although peak‐ enhancement occurred earlier for nine of ten ductal carcinomas, this difference was not significant (*P* = 0.8, Table 1). There were no statistically significant differences in IQR for T_1pre,_ [Gd]_peak,_ [Gd]_post,_ %ER_peak_, and %ER_post_ between the two cancer groups (*P* > 0.05, *P* values in Table 2), but the T_1_ IQR was significantly higher for lobular carcinomas in peak‐ and post‐enhancement frames (*P* < 0.05, *P* values in Table 2).

**Table 1:** Mean ± standard deviation of median T_1_, [Gd], and %ER across the different time points and median peak‐enhancement frame for the lobular and ductal cancer groups

Tumor group	T_1pre_ (ms)	T_1peak_ (ms)	T_1post_ (ms)	[Gd]_peak_ (×10^−4^ mmol/ml)	[Gd]_post_ (×10^−4^ mmol/ml)	%ER_peak_	%ER_post_	Peak‐enhancement frame
Lobular carcinomas (n = 10)	1275 ± 623	587 ± 367	591 ± 363	3.9 ± 2.2	3.9 ± 2.3	116 ± 33	114 ± 33	5 (4–8)
Ductal carcinomas (n = 10)	1087 ± 471	477 ± 193	494 ± 201	3.9 ± 1.9	3.7 ± 1.7	118 ± 32	111 ± 27	6 (3–8)
*P*‐value	0.9	1	1	0.8	1	1	1	0.8

**Table 2:** Mean ± standard deviation of IQR T_1_, [Gd], and %ER for the lobular and ductal cancer groups across the different time points

Tumor group	IQR T_1pre_ (ms)	IQR T_1peak_ (ms)	IQR T_1post_ (ms)	IQR [Gd]_peak_ (×10^−4^ mmol/ml)	IQR [Gd]_post_ (×10^−4^ mmol/ml)	IQR %ER_peak_	IQR %ER_post_
Lobular carcinomas (n = 10)	388 ± 251	206 ± 110	200 ± 118	2.1 ± 1.3	2.4 ± 2.4	62 ± 29	57 ± 25
Ductal carcinomas (n = 10)	251 ± 110	108 ± 39	113 ± 48	1.5 ± 0.7	1.5 ± 0.7	49 ± 12	48 ± 10
*P*‐value	0.14	**0.02**	**0.04**	0.58	0.77	0.43	0.68

Bold values indicate significant differences.

**Figure 5:**
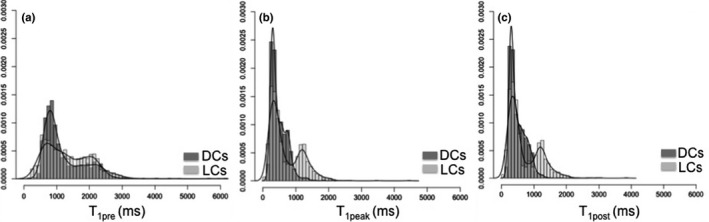
Lobular carcinoma (LC) and ductal carcinoma (DC) T_1_ distributions with the corresponding distribution lines for the pre‐, peak‐, and post‐contrast phases. The bimodal distribution of the post‐enhancement T_1_ values observed for LC simply shows the uptake variation in the analyzed cases.

Global T_1_ histograms (100 ms bin size) for the pre‐, peak‐, and post‐contrast enhancement frames are shown in Fig. 5. The number of voxels in the first bin (T_1_ ≤ 100 ms) falling to the nonlinear part of the calibration curves (Fig. 1) was 0, 18, and 16 for T_1pre_, T_1peak_, and T_1post_, that is, no more than 0.4% of the total number of tumor voxels (4029). Therefore, the obtained T_1_ values demonstrate that the dynamic range of our DCE sequence suits the range of the T_1_ values measured in clinical examinations before and after contrast administration; the range of the T_1_ values for our cohort falls within the range, for which the image intensity is approximately directly proportional to 1/T_1_ (or R_1_) for our DCE sequence (Fig. 1). The distribution of the native T_1_ values (T_1pre_) is similar between lobular and ductal carcinomas [Fig. 5(a)], whereas the distributions of the T_1_ values after the contrast administration (T_1peak_ and T_1post_) suggest greater enhancement variability for the lobular carcinomas [Figs. 5(b) and 5(c)].

## 4 Discussion

The value of quantitative measurements in breast has already been demonstrated in the context of pharmacokinetic modeling. Using a small number of patients, we demonstrated a method to assess T_1_ in clinical fat‐suppressed DCE‐MRI examinations, thus quantifying contrast agent uptake. The proposed quantitative approach broadens the scope of the clinical DCE examination, is practical and achievable on any clinical MRI system, as it simply requires a calibration with test objects. At this stage, the T_1_ calculations are performed off‐line, but they could be easily integrated as a postprocessing step. The use of quantitative methods enables direct comparisons of examinations in a longitudinal patient study or examinations from different patients; quantitative parameters such as T_1_ (pre‐ and post‐contrast) and contrast agent concentration can be measured separately. The semiquantitative enhancement ratio, in contrast, is affected by both T_1_ and contrast‐agent concentration and is therefore more difficult to interpret signal changes relate to the baseline image intensity in a T_1_‐weighted acquisition.

In addition to proposing and demonstrating a novel approach to quantitative breast MRI, we also demonstrated that the dynamic range of our DCE pulse sequence is suitable for our clinical workload; the image intensity is approximately directly proportional to 1/T_1_ over the range of T_1_ values we measured in breast lesions. Considering that the national guidelines for the breast screening program require that the contrast characteristics of the DCE sequence are evaluated,^10^ it is essential to provide methods to do so. To the best of our knowledge, this article is the first to demonstrate that the DCE contrast characteristics are correct for the actual range of T_1_ values found in clinical practice within our patient population, taking into account specific constraints such as the rate of injection, the contrast agent dose and type, for example, which may vary in different populations.

Inaccurate T_1_ measurements can be caused by the spatial variation in flip angle as a result of B_1_ field inhomogeneity^13^ and inaccurate RF transmitter power calibration. The use of uniformity filters in clinical examinations is also a factor that could potentially introduce errors, decreasing the level of confidence in quantification studies. These filters have shown to alter noise distribution resulting in SNR changes.^14^ Although B_1_ inhomogeneity is more pronounced at higher fields (≥ 3 T), all the aforementioned sources of error affect both standard T_1_ calculations in pharmacokinetic modeling and fat‐suppressed T_1_ measurements. B_0_ variations are an additional issue, specific to breast DCE with fat suppression; good B_0_ homogeneity is required to avoid suppression of water signals. In this study, these main factors to affect accuracy of the T_1_ values obtained with the proposed method were investigated thoroughly. Calibration curves proved to be highly reproducible and a good agreement between IR and fat‐suppressed T_1_ measurements was demonstrated; the discrepancy found for very long T_1_ values is probably due to a lower SNR. Spatial variation of the B_1_ field was investigated with a uniform test object. B_1_ inhomogeneity and the use of uniformity filters were found not to affect significantly the T_1_ calculation over the breast volume for our 1.5 T system in a conductive test object. B_0_ inhomogeneity was simulated altering the central frequency. Small frequency variations introduce calibration errors if the water signal is suppressed, but unintentional water suppression is relatively rare.^15^ Recent developments in shimming are encouraging^16^ and will in general lead to improved performance in commercial systems. A separate issue is that DCE images may have fat and water out of phase; in case of fat suppression failure, no quantitative measurements are possible for voxels containing both fat and water. Nevertheless, the measurements of T_1_ on breast lesions are likely to be less affected by fat suppression failure than the measurements on breast parenchyma, as breast tumors are not expected to have a significant fat content.

Comparing lobular carcinomas and invasive ductal carcinomas, we found later enhancement for lobular carcinomas in our cohort, in accordance with previous studies,^8^, ^9^ but these differences were not statistically significant. Also, similar peak‐enhancement was found for the two cancer groups, in agreement with Mann et al.^8^ T_1_ relaxation time is tissue specific and having a quantitative method to measure it allowed the analysis to go further and interpret the distribution of the T_1_ values for the lobular and ductal carcinomas. Significant differences in the IQR for T_1peak_ and T_1post_ between the two patient groups were detected, potentially reflecting their distinct growth and invasion patterns. Lobular cancers may grow in a loosely cohesive manner invading surrounding tissue, whereas ductal cancers usually follow a self‐contained solid growth pattern.^17^ Ductal carcinomas are therefore more likely to present similar characteristics within a patient population. Figure 4 aims to demonstrate that contrast agent uptake is not necessarily proportional to ER, which is only semiquantitative. In only one of the cases presented, contrast agent uptake rises approximately monotonically with increasing ER, and this could be attributed to differences in the baseline T_1_ values between the two tumors. However, the effects of noise and their dependence on T_1_ cannot be excluded. The supplementary figure also demonstrates the relationship between ER and [Gd] post‐enhancement for lesions with different native T_1_ values.

We acknowledge the limitations of this study on a small number of subjects; however, our scope was to demonstrate the potential of the proposed approach. We detected significant differences in the range of T_1_ values post‐contrast between lobular and ductal carcinomas, but no significant differences between the range of contrast agent concentration values or enhancement ratio values. These findings merit further investigation, as T_1_ values could be proposed as independent biomarkers and be directly related to other tumor characteristics in larger cohorts.

Although the sequences employed complied with the DCE‐MRI national guidelines for temporal resolution, we cannot exclude that some variations observed might be system and protocol dependent. Qualitative assessment of the enhancement curves may be reader dependent leading to inconsistent interpretation of uptake in tumors.^3^ In quantitative studies, many variations can also arise from different MR systems and DCE sequence parameters.^18^, ^19^, ^20^, ^21^ Ledger et al. highlighted the effect of flip angle and k‐space sampling on fat suppression efficiency, dynamic range, and therefore the relationship between signal intensity and 1/T_1_ for the range of the expected T_1_ values.^12^ Therefore, the proposed method for quantitative T_1_ measurements in fat‐suppressed DCE may also need to be validated for other sequence parameters and in other systems. Further work is currently in progress.

In conclusion, fat‐suppressed T_1_ measurements are viable in breast DCE, resulting in quantitative measurements of contrast agent uptake. The proposed quantitative approach enables the optimization of DCE protocols by tailoring the dynamic range of the sequence to the values of T_1_ measured for each population. T_1_ measurements from clinical fat‐suppressed DCE demonstrated the variations in the T_1_ range between ductal and lobular cancer within a relatively small number of patients. This work has demonstrated the feasibility of fat‐suppressed T_1_ measurements as a tool for clinical studies.

## Supporting information


**Fig. S1.** %Enhancement Ratio versus [Gd] and native T1 distribution for different lesions.

## Figures and Tables

**Figure A1:**
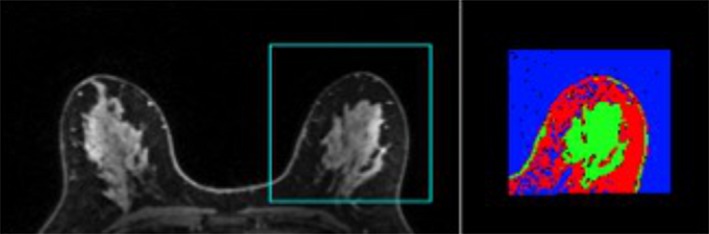
Segmentation of the contralateral breast of a patient with invasive lobular carcinoma on the right breast. Within ROI, three clusters were assigned to fat (red), fibroglandular tissue (green) and background (blue). [Color figure can be viewed at wileyonlinelibrary.com]

## Appendix 1

### 1.1 T_1_ measurements on radiologically normal fibroglandular tissue.

The purpose of this section is to provide further evidence to support the clinical use of our proposed method for T_1_ measurements. The radiologist's reports on the contralateral side of the 20 tumours considered in this study were scrutinized, and the radiologically normal breasts (n = 14) were selected. The method proposed in this article to calculate T_1_ relaxation time was applied to the normal‐appearing contralateral breast fibroglandular tissue. The remaining 6 patients had multifocal disease, benign lesions or random findings on their contralateral breast and therefore were excluded from the analysis. The fibroglandular tissue was segmented on a central PD image by applying clustering segmentation (IDL 8.4 Boulder, CO, USA) to a manually selected ROI containing the contralateral breast (Fig. A1).

The segmented DCE and PD datasets were combined and the T_1_ was calculated on a pixel‐by‐pixel basis before contrast administration, using the test‐object calibration curves as described in the Methods section. Table A1 reports the median T_1_ of the contralateral fibroglandular tissue for each patient. The average T_1_ is 947 ± 310 ms (mean ± standard deviation).

**Table A1:** Median T_1_ values of the contralateral normal‐appearing breast tissue

Subject	Median T1 (ms)
1	1036
2	1130
3	1319
4	1069
5	751
6	635
7	757
8	507
9	1501
10	629
11	881
12	952
13	689
14	1410

The calculated values using the proposed approach are close to previously reported values at 1.5T by Rakow‐Penner et al. They employed inversion‐recovery to measure the T_1_ of normal fibroglandular tissue on regions with high tissue homogeneity.^22^ The subjects of our study, however, cover a wide range of ages, hormonal status and were not scanned in the same hormonal phase. Although our measurements include the entire fibroglandular tissue on a central slice, thus considering tissue density variations within the breast, the broad agreement with the literature^22^, ^23^, ^24^, ^25^ supports the clinical use of our method.
