# Supplementary figures and images for: Quantitative evaluation of contrast agent uptake in standard fat‐suppressed dynamic contrast‐enhanced MRI examinations of the breast
Source: Med Phys. 2017 Nov 30;45(1):287–96. doi: 10.1002/mp.12652 (PMC5814859; doi:10.1002/mp.12652)

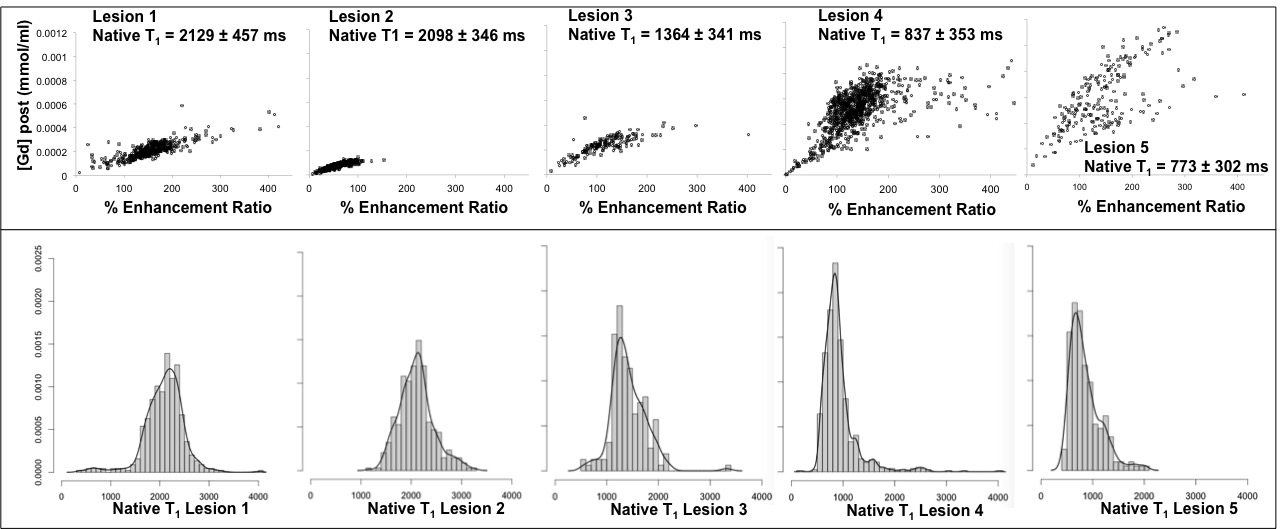

Supplement: Supplementary file 1 — Fig. S1. %Enhancement Ratio versus [Gd] and native T1 distribution for different lesions. [file MP-45-287-s001.tif]
